# Smart Monitoring and Control in the Future Internet of Things

**DOI:** 10.3390/s22010027

**Published:** 2021-12-22

**Authors:** Franco Cicirelli, Antonio Guerrieri, Andrea Vinci

**Affiliations:** Institute for High Performance Computing and Networking (ICAR), Italian National Research Council (CNR), 87036 Rende, Italy

The Internet of Things (IoT) and related technologies are promising in terms of realizing pervasive and smart applications, which, in turn, have the potential to improve the quality of life of people living in a connected world. According to the IoT vision, all things can cooperate with each other and can be managed from anywhere via the Internet to allow tight integration between physical and cyber worlds, thus improving efficiency, promoting usability, and opening up new application opportunities. Today, IoT technologies are successfully exploited in several domains, providing both social and economic benefits. The realization of the full potential of the next generation of the Internet of Things still needs further research efforts concerning, for instance, the identification of new architectures, methodologies, and infrastructures dealing with distributed and decentralized IoT systems; the integration of the IoT with cognitive and social capabilities; the enhancement of the sensing–analysis–control cycle; the integration of consciousness and awareness in IoT environments; and the design of new algorithms and techniques for managing IoT big data.

This Special Issue has gathered contributions and research efforts about advancements in the IoT domain covering important topics such as networking and communication; frameworks and platforms; approaches for modeling, information analysis, and discovery; and indoor localization and tracking. Research outcomes have been provided in the fields of smart environments, smart manufacturing, smart health, and smart infrastructures.

This Special Issue includes twelve papers that offer interesting perspectives on the field, covering most of the mentioned topics. The issue opens with the paper “Approaching the Communication Constraints of Ethereum-Based Decentralized Applications” [1], in which authors apply blockchain principles to the IoT. Such a topic is challenging due to the constraints of IoT devices. These constraints are presented by the authors from the perspective of end devices in the development of IoT applications based on the Ethereum blockchain, which is one of the viable and very popular platforms for IoT blockchain applications. Moreover, the authors elaborate and compare in terms of computation and communication constraints, as well as in terms of security, three architectural approaches for the design of IoT end-device applications based on the Ethereum blockchain. Finally, the results of communication-traffic measurements in these architectures clearly estimate the communication constraints.

The second contribution [2], titled “Computation of Traffic Time Series for Large Populations of IoT Devices”, starts from the concept that the correct behavior of IoT devices can be monitored by inspecting the traffic that they create. In this paper, the authors sustain that this passive monitoring methodology allows the detection of device failures or security breaches. Such monitoring creates a huge amount of traffic (in time-series) that is not manageable without highly optimized algorithms. In the paper, the authors compare three algorithms for time-series extraction from traffic captured in real-time, demonstrating that a single-core central processing unit (CPU) can extract more than three bidirectional traffic time series for each one of more than 20,000 IoT devices in real-time using the *DStries* algorithm with recursive search. Finally, the proposed approach also enables a fast system reconfiguration when new IoT devices are added to the network.

The paper in [3], namely “A Feature-Based Model for the Identification of Electrical Devices in Smart Environments”, starts from the idea that data mining and analysis of energy data of electrical devices in a Smart Home is very important for the decision-making process. In particular, this is true for energy management, both from the consumer perspective (who wants to save money) and also in terms of energy redistribution and reduction of carbon dioxide emission. In this context, advanced monitoring and control mechanisms are necessary to deal with the identification of appliances. For this reason, the authors of this work propose a model for appliances’ automatic identification. Such a model is based on a set of 19 features that are extracted by analyzing energy consumption, time usage, and location from a set of device profiles. Moreover, machine learning approaches are employed by experimenting with different classifiers based on such a model for the identification of appliances.

The paper “Development and Application of an Atmospheric Pollutant Monitoring System Based on LoRa—Part I: Design and Reliability Tests” [4] proposes the design and development of an embedded system devoted to monitoring atmospheric pollutant concentrations. The system exploits LoRa wireless communication technology, which is one of the most used technologies for the IoT. The proposal has been designed to be modular in terms of both hardware and software components so as to foster later system expansions. Separation of concerns is also exploited. In particular, splitting each function into independent modules permits fast system development and increases system stability. The system proves its effectiveness and soundness for implementing applications concerning real, remote, atmospheric-pollutant-concentration monitoring with the specific requirements for the intended application environment. The actual application also demonstrates that the proposal has strong market prospects.

The paper “A Quantum Ant Colony Multi-Objective Routing Algorithm in WSN and Its Application in a Manufacturing Environment” [5] highlights some limitations in the traditional routing algorithms for WSNs. In particular, the focus is on ant-based routing algorithms that, in their basic formulations, can easily fall into a “local optimum”, thus leading to premature stagnation of the algorithms. In order to overcome the limitations of ant colony optimization (ACO)-based routing methods, this paper introduces the idea of using quantum-inspired evolutionary algorithms and ACO together. This approach promotes the balancing of loads, real-time transmission, and energy consumption by using a multi-objective fitness function. In this direction, the authors propose a Quantum Ant Colony Multi-Objective Routing (QACMOR) algorithm, which is a new, yet efficient, routing approach for WSNs with the potential for solving large-scale routing problems. In the proposal, quantum bits are used to represent the node pheromone, and quantum gates are used to update the pheromone of the search path.

In [6], a paper named “A Posture Recognition Method Based on Indoor Positioning Technology” is presented. This paper introduces a new schema for body posture recognition, which is based on indoor positioning technology. The authors deployed an indoor positioning system by equipping people with wearable receiving tags. These tags were purposely deployed at some specific key points of the human body. In the paper, the method for measuring tag distances relies on ultra-wideband radio technology. Measuring such distances allows understanding human postures. In the posture-recognition algorithm, least-square estimation method and the improved extended Kalman filtering algorithm are respectively adopted to suppress the noise of the measurement of the distances and to improve the accuracy of positioning and recognition. Some simulation results show that the implemented approach is more effective in error performance with respect to the state of the art.

The manuscript “A Novel Passive Indoor Localization Method by Fusion CSI Amplitude and Phase Information” [7] presents FapFi, which is a passive indoor fingerprint system based on WiFi using the fusion amplitude and phase information of the Channel State Information (CSI) signal. FapFi is able to detect the anomalous CSI amplitude values at the subcarrier level as well as the redundant values at the channel level. The amplitude information is filtered, and a linear transformation is then applied to extract the calibrated phase information. Finally, the fusion of the feature information is stored in a fingerprint database as a basis for indoor positioning. The approach is able to provide robust fingerprint features while reducing the signal interference from environmental factors and to fulfill real-time localization requirements for passive human indoor localization with high-precision positioning. Furthermore, the authors investigate the main parameters affecting the performance of the positioning accuracy.

“A Non-Intrusive Approach for Indoor Occupancy Detection in Smart Environments” is the paper presented in [8]. The main scope of the paper is the design and development of an affordable and non-intrusive solution devoted to improving dwellers’ experience in Smart Environments through the use of ML algorithms. Data about temperature, light intensity, noise, and CO_2_ are gathered to estimate the presence of occupants. The proposed approach was purposely designed so as to support a fast integration with other complementary solutions known in the literature. The proposal first has the aim to collect and analyze environmental data and then to apply ML techniques to infer the occupancy of the area under monitoring. The proposed solution was tested in a real-world scenario using a prototype system. The obtained results demonstrate the effectiveness and usability of the proposed approach for occupancy detection in indoor environments.

The next contribution of the Special issue is reported in [9] and is titled “Computational Efficiency-Based Adaptive Tracking Control for Robotic Manipulators with Unknown Input Bouc–Wen Hysteresis”. The paper investigates an unknown input Bouc–Wen hysteresis control problem for robotic manipulators. An adaptive control and a dynamical gain-based approach are used. Moreover, an additional control unit is used to model the dynamics of hysteresis in the closed-loop system, which are then integrated with the robotic manipulators. Two adaptive parameters are introduced for improving the computational efficiency of the proposed control scheme. Using these parameters, the outputs of robotic manipulators are driven to track desired trajectories. In this context, Lyapunov theory is adopted to prove the effectiveness of the proposed method. Furthermore, the tracking error is reduced from ultimately bounded to asymptotic tracking compared to most of the existing results. This is of significance to improve the control quality of robotic manipulators with unknown input Bouc–Wen hysteresis. The authors provide some results, including fixed-point and trajectory controls, to show the soundness of the proposed approach.

The paper in [10], named “An IoT Platform with Monitoring Robot Applying CNN-Based Context-Aware Learning”, proposes an IoT-based platform with an intelligent surveillance robot using machine learning. The goal is that of overcoming some limitations of the existing fixed closed-circuit television (CCTV). The IoT platform coupled with a surveillance robot provides an active CCTV by realizing smart monitoring. The smart system can realize line tracing and acquire contextual information through some sensors to detect abnormal status of an environment. Moreover, the manager of the system can receive an alarm through a smartphone if an abnormal status on the monitored environment is detected. Furthermore, the end user of the system is equipped with an app to remotely control the robot. The context-aware information is extracted using convolutional neural network (CNN)-based algorithms. Experimental results show very high accuracy of the learning process. A further contribution of the paper consists in the construction of a CNN model for image-and-context-aware learning and intelligence enhancement of the proposed IoT platform. The proposed IoT platform can be used to catch abnormal status in various industrial fields such as factories, smart farms, logistics warehouses, and public places.

In [11], the paper “An Integrative Framework for Online Prognostic and Health Management Using Internet of Things and Convolutional Neural Network” is presented. Here, the authors face the lack of a systematic and comprehensive framework of prognostic and health management (PHM) in the context of manufacturing enterprises. In this direction, they propose an integrative framework to systematically solve the problem from three perspectives, namely strategic, tactical, and operational. At the strategic level, an innovative business model to create added value through big data is provided; the tactical level furnishes, through a convolutional neural network, an implementation route for application integration, analysis service, and visual management so as to satisfy various functional requirements from different stakeholders; the operational level builds a self-sensing network based on anti-inference and self-organizing Zigbee to capture the real-time data from the equipment group. Finally, the feasibility of the framework is verified in a real case study.

The last paper of the special issue is “Landslide Susceptibility Assessment Using Integrated Deep Learning Algorithm along the China–Nepal Highway” [12]. It starts from important considerations about the China–Nepal Highway, which is a vital land route in the Kush–Himalayan region suffering serious concerns due to the occurrence of mountain hazards. Based on temporal and spatial sensor data, the paper leverages data-driven algorithms to predict landslide vulnerabilities. In particular, authors consider ten landslide instability factors for their study, such as elevation, slope angle, slope aspect, plan curvature, vegetation index, built-up index, stream power, lithology, precipitation intensity, and cumulative precipitation index. Starting from these data, four machine learning algorithms (i.e., decision tree, support vector machines, Back Propagation neural network, and Long Short Term Memory) are exploited and their performance compared. The experimental results show that the Long Short Term Memory algorithm outperforms the other three models due to its ability to learn time series with long temporal dependencies. As a final remark, the study highlights that the dynamic change course of geological and geographic parameters is an important indicator in reflecting landslide susceptibility.
